# Use of Psychotropic Drugs during the COVID-19 pandemic in Minas Gerais, Brazil

**DOI:** 10.1590/1980-549720230059

**Published:** 2023-12-11

**Authors:** Juliana Cerqueira Barros, Sarah Nascimento Silva

**Affiliations:** I Universidade Federal de Minas Gerais, Pharmacy School – Belo Horizonte (MG), Brazil.; II Fundação Oswaldo Cruz, Instituto René Rachou, Health Technology Assessment Unit – Belo Horizonte (MG), Brazil.

**Keywords:** Psychotropic drugs, Mental health, COVID-19, Pharmacoepidemiology, Pharmaceutical services, Database, Psicotrópicos, Saúde mental, COVID-19, Farmacoepidemiologia, Assistência farmacêutica, Base de dados

## Abstract

**Objective:**

To describe the profile of dispensation of mental health drugs by analyzing trends in use before and during the COVID-19 pandemic within the Unified Health System (Sistema Único de Saúde [SUS]).

**Methods:**

Pharmacoepidemiological study based on the retrospective analysis of records regarding the dispensation of psychotropic medicines in the SUS database in the state of Minas Gerais between 2018 and 2021, considering the periods before (2018–2019) and during the COVID-19 pandemic (2020–2021). A database with the records of dispensation of municipalities was created, and the consistency of releases was verified using the Analysis of Variance (ANOVA) test. Medicine consumption was measured in a defined daily dose (DDD) per 1,000 inhabitants/day for SUS, and the difference between periods was evaluated using Student's t-test.

**Results:**

During the COVID-19 pandemic, there was an increase in the consumption of psychotropic drugs in SUS-MG. The most consumed medicines were fluoxetine hydrochloride, diazepam and phenobarbital sodium (DDD=5.89; 3.42; 2.49) in the Basic Pharmaceutical Services Component(CBAF), and olanzapine, risperidone and quetiapine hemifumarate (DDD=0.80; 0.47; 0.38) in the Specialized Pharmaceutical Services Component (CEAF). The highest percentage increase in consumption was attributed to clonazepam (75.37%) and lithium carbonate (35.35%), in CBAF, and levetiracetam (3,000.00%) and memantine hydrochloride (340.0%) in CEAF.

**Conclusion:**

The change in the psychotropic drug dispensation profile during the COVID-19 pandemic highlights the need to produce more studies to complete, confirm or rule out this profile and monitor the use of psychotropic drugs by the population in the post-pandemic context.

## RESUMO

**Objetivo:**

Descrever o perfil de dispensação de medicamentos da saúde mental analisando o uso antes e durante a pandemia de COVID-19 no âmbito do Sistema Único de Saúde (SUS).

**Métodos:**

Estudo farmacoepidemiológico a partir da análise retrospectiva dos registros de dispensação de psicofármacos na base de dados do SUS no estado de Minas Gerais (MG) nos períodos antes (2018–2019) e durante a pandemia de COVID-19 (2020–2021). Um banco de dados com os registros de dispensação dos municípios foi elaborado, sendo verificada a consistência de lançamento pelo teste de Análise de Variância (ANOVA). O consumo dos medicamentos foi mensurado em dose diária definida (DDD) por 1.000 habitantes/dia para o SUS, sendo a diferença entre os períodos avaliada pelo teste estatístico t de Student.

**Resultados:**

Durante a pandemia de COVID-19 houve aumento no consumo de psicofármacos no SUS-MG. Os medicamentos mais consumidos foram cloridrato fluoxetina, diazepam e fenobarbital sódico (DDD=5,89; 3,42; 2,49) no componente básico (CBAF), e olanzapina, risperidona e hemifumarato de quetiapina (DDD=0,80; 0,47; 0,38) no componente especializado da Assistência Farmacêutica (CEAF). Os maiores aumentos percentuais no consumo foram atribuídos ao clonazepam (75,37%) e carbonato de lítio (35,35%) no CBAF e levetiracetam (3.000,00%) e cloridrato de memantina (340,00%) no CEAF.

**Conclusão:**

A alteração do perfil de dispensação de psicotrópicos durante a pandemia de COVID-19 alerta quanto à necessidade de produção de mais estudos a fim de completar, confirmar ou afastar este perfil e monitorar o uso de psicofármacos pela população no contexto pós-pandêmico.

## INTRODUCTION

The availability of information based on valid and reliable data allows an objective analysis of the sanitary situation of a region, being a source of information for decision making and the schedule of strategic actions^1^. In this context, many databases can contribute with an important tool to verify health indicators. The access to essential medicines and the profile of use by the population constitute important Pharmaceutical Services indicators, which are measured and monitored in several countries and can express access, quality and organization, in terms of structure and processes, of the services provided for the population^2^.

In 2022, about one billion people were affected by a diagnosable mental disorder; however, only a small fraction of them had access to efficient, accessible and qualified care^3^. After 2020, with the COVID-19 pandemic, many studies started to report a substantial increase in depressive (28%) and anxiety (26%) disorders in relation to the previous year^3,4^. Many of these disorders were attributed to several short or long-term stressors which led to the development or worsening of disorders in the mental health field^3,5^, such as the social isolation and contingency actions^3,6^, uncertainties about the virus and information overload, besides the stress caused by unemployment and financial insecurity^8^. Therefore, the insurance of adequate access to treatment, the rational use of medicines and their special control according to the current sanitary regulations are extremely relevant tasks for the management of Pharmaceutical Services.

In 2020, many countries made efforts to develop or adjust psychological interventions in their national plans in order to treat or prevent mental health conditions as a response to COVID-19^9^. In Brazil, the increase of care to health professionals and financial transfers to cities were identified in the first year of the pandemic^10^. In the scope of Pharmaceutical Services, many general actions were proposed to readjust the Unified Health System (SUS) services, so as to supply health technologies with sustainability and promote the rational use of these resources in health care^11^. Temporary changes in the legislation that regulates the dispensation of medicines extended the expiration dates of prescriptions and the periods to return to the drugstores, thus increasing the quantity of dispensed medications in each period. This data can be verified in the records of administrative databases^12^.

Psychotropic drugs are controlled medicines that can cause physical and psychological dependence, with major adverse events^3^. The use of antipsychotic drugs is growing in Brazil; a national research indicated that 8.7% of adults in the country use at least one psychotropic drug^13^, and many studies show important prevalence in the use of psychotropic drugs in different Brazilian regions^14,15^. The increase in sales of psychotropic drugs in Brazil during the COVID-19 pandemic^16^, the temporary change of dispensation rules for these medicines^12^ and the growing phenomenon of health medicalization^17^ are situations that can contribute with the inadequate or irrational use of drugs, which requires an investigation to understand and address the attention to individuals with mental disorders. Studies about the access and use of drugs during the COVID-19 pandemic using the administrative databases of SUS can be an important source of information to observe new trends and the impact of the emergency actions and measures in this period. This study aims at describing the profile of dispensation of psychotropic drugs analyzing their use before and during the COVID-19 pandemic in the scope of SUS.

## METHODS

### Type of study and location

This is a pharmacoepidemiological study, based on a retrospective, descriptive and quantitative analysis of secondary data of drug dispensation records from SUS in the state of Minas Gerais, the Integrate System of Pharmaceutical Services Management (SIGAF). SIGAF is an administrative governmental base for the record and book-keeping of dispensation of medicines in all of the 853 cities of the State, representing an essential system for the integration of SUS in the State, considering that it supports and subsidizes the performance of activities and processes developed in the drugstores of each city, which increases the effectiveness and the management of logistics processes^18^.

### Participants

The study population includes all users registered in the SIGAF system who acquired any psychotropic drug between January 2018 and December 2021. This period was chosen for contemplating two different moments: “before the COVID-19 pandemic” (January 2018 to December 2019) and “during the COVID-19 pandemic” (January 2020 to December 2021). The data were obtained based on the reports of medication dispensation registered in SIGAF, attributed to the Regional Health Superintendency and eight Regional Health Administrations, made available in an anonymous manner through a request made in the Transparency Portal of the government of Minas Gerais (Protocol: 01320000052202240). The records of dispensation of medicines present in the report are directly related to the patients in dispensation units, and do not include those used in health services such as hospitals, urgency and emergency care and outpatient clinics. The service records allow to enter more than one monthly service per patient, whose control is ruled by medical prescription. There was no crossing of data to identify the patients, given the limitation of data anonymization available for analysis.

### Sources of data and analysis

A database with the records of dispensation of psychotropic drugs was elaborated. The medicines selected to compose the database are the ones present in the National Relation of Essential Medicines (Rename) of 2022 with indication for mental health treatment. This indication was verified in the National Therapeutic Form (FTN), for drugs allocated in the Basic Pharmaceutical Services Component (CBAF), or in the Clinical Protocols and Therapeutic Guidelines (PCDT) (Aggressive Behavior in Autism Spectrum Disorder; Alzheimer's Disease; Schizophrenia; Bipolar Disorder; and Schizoaffective Disorder) for medications allocated in the Specialized Pharmaceutical Services Component (CEAF). A list of psychotropic drugs selected for analysis was elaborated for each component, being grouped by annual records and, then, by active ingredient, identifying the dispensed quantities. The data were tabulated using the Microsoft Excel in order to group the records per city. An analysis of the frequency of monthly entries of dispensation records by the cities that are part of SIGAF was performed to identify sources of bias coming from the irregularity in the registration of data, using descriptive statistics and the Analysis of Variance (ANOVA). This analysis only contemplated the records of medicines in CBAF, considering that dispensations of CEAF are concentrated in units whose use of SIGAF is mandatory. The description of medicines and active ingredients was standardized manually to minimize the duplicity of items with the same description.

The variables of interest include the dispensed quantities, expressed in pharmaceutical units (pills, capsules or ampoules), in cardinal numbers, and in a defined daily dose per 1,000 inhabitants/day (DDD), established by the WHO, being corrected for the size of the population that uses SUS, that is, 75% of the population in the state of Minas Gerais. Besides, the number of services was described based on the monthly reports of SIGAF, identifying time series for each one of the medicines, gathered by active ingredient and component of Pharmaceutical Services. The temporal analysis contemplated not Only the annual data, but the periods “before the COVID-19 pandemic” and “during the COVID-19 pandemic”, defining two sets of data for comparison. The analysis of the dispensation records was performed by descriptive statistics expressing measures of central tendency (mean and median), and measures of variation for the records. The difference between dispensations, taken as the calculated consumption in DDD, during the two analyzed periods, was assessed by the Student's t-test considering the consumption metrics as the dependent variable, and a 95% confidence level. This study's report adopted the checklist *Reporting of studies Conducted using Observational Routinely collected health Data* (RECORD)^19^.

This study includes research with databases, whose information is aggregated, without the possibility of individual identification, being dismissed from submission to the Ethics Committee, as established by resolution n. 510/2016 of the Brazilian National Health Council.

## RESULTS

**Figure 1:**
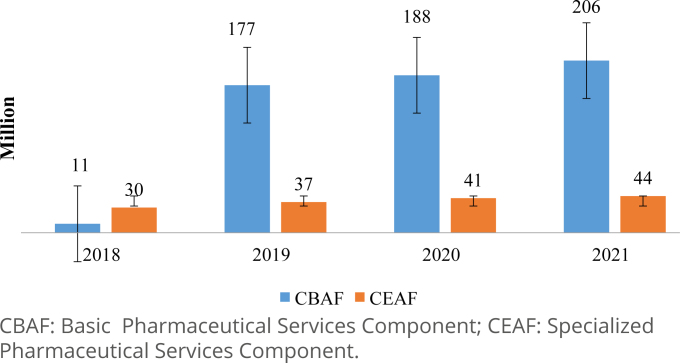
Pharmaceutical units of psychotropic drugs dispensed in the Unified Health System of Minas Gerais in 2018–2021.

The reports extracted from SIGAF present 13.9 billion records of dispensation to assist 94.9 million patients between 2018 and 2021. The dispensations referring to mental health drugs correspond to 15.7% of all records in SIGAF's database, providing for 10.7% of the patients. A growing tendency was observed in the number of pharmaceutical units dispensed annually, both for drugs in CBAF and CEAF (Figure 1).

The analyzed records of dispensation include the data entry of 834 cities registered in SIGAF, localized in 28 health regionals in the state of Minas Gerais, which represents 99.7% of the 853 cities in MG. The frequency of data entry in SIGAF's database by the cities of the State is different every year (p<0.001); however, this variation presents low amplitude (1.80–5.16%), indicating the continuous data entry in SIGAF's database by the registered cities in the analyzed period.

Forty-six drugs used for mental health treatments were identified in the scope of CBAF. The analysis of the records of medication dispensation in SIGAF's database identified a series of data of 38 drugs from 2018 to 2021, representing 16 active ingredients. The consumption of flumazenil was not calculated due to the absence of the DDD/ATC classification. Forty drugs used in mental health treatments were identified in the scope of CEAF, and all of them had records of dispensation in SIGAF's base, representing 17 active ingredients.

**Figure 2:**
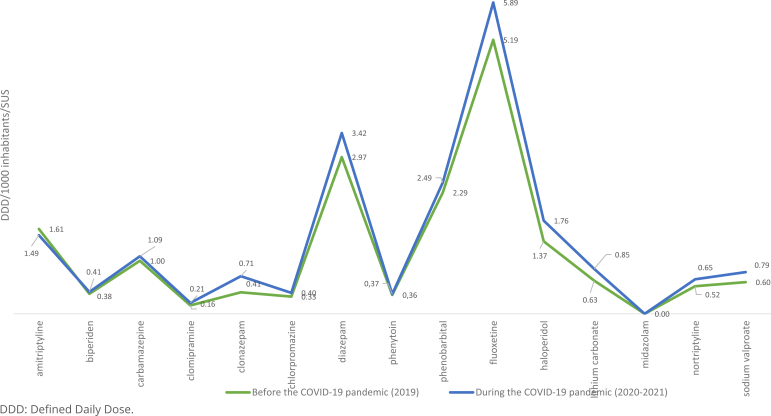
Consumption in Defined Daily Dose/1000 inhabitants/day of psychotropic drugs in the Basic Pharmaceutical Services Component in the Unified Health System of Minas Gerais, 2019-2021.

The analysis of records referring to CBAF points out that, during the pandemic (2020-2021), fluoxetine hydrochloride was the most dispensed medicine (mean DDD=5.89), followed by Diazepam (mean DDD=3.42), phenobarbital sodium (mean DDD=2.49) and haloperidol (mean DDD=1.76) (Figure 2).

The regionals that most dispensed medicines related to mental health in CBAF were, respectively, Divinópolis (12.24%), Belo Horizonte (7.49%), Sete Lagoas (5.38%), Ponte Nova (4.93%) and Patos de Minas (4.90%). The number of records of dispensation in 2018 presented a disagreeing number, much lower than the one observed in 2019, 2020 and 2021. Therefore, in the comparative analysis of the historical series, the choice was to consider the data after 2019. The comparative analysis between periods, before and during the COVID-19 pandemic, showed significant statistical differences between each period (p=0.009), with the increasing consumption of all psychotropic drugs in CBAF during the pandemic, except for amitriptyline, which presented reduction in consumption (-7.92%). Clonazepam and lithium carbonate presented the highest percentage increase of consumption during the pandemic: 75.37 and 35.35%, respectively (Table 1).

**Table 1:** Comparison of the consumption of psychotropic drugs in the Basic Pharmaceutical Services Component in Minas Gerais, 2018–2021.

Active ingredient	DDD/1000 inhabitants/day/SUS									
	2018	2019	2020	2021	Mean	Median	Before the COVID-19 pandemic (2019)	During the COVID-19 pandemic (2020–2021)	Absolute variation	Percentage variation (%)
Amitriptyline	0.06	1.61	1.43	1.55	1.16	1.49	1.61	1.49	-0.12	-7.35
Biperiden	0.00	0.38	0.40	0.42	0.30	0.39	0.38	0.41	0.04	9.26
Carbamazepine	0.90	1.00	1.03	1.15	1.02	1.02	1.00	1.09	0.09	8.47
Clomipramine	0.01	0.16	0.20	0.22	0.15	0.18	0.16	0.21	0.05	29.56
Clonazepam	0.00	0.41	0.43	0.99	0.46	0.42	0.41	0.71	0.31	75.37
Chlorpromazine	0.08	0.33	0.38	0.41	0.30	0.35	0.33	0.40	0.07	21.54
Diazepam	0.03	2.97	3.41	3.44	2.46	3.19	2.97	3.42	0.46	15.41
Phenytoin	0.04	0.36	0.36	0.39	0.29	0.36	0.36	0.37	0.02	4.20
Phenobarbital	0.23	2.29	2.60	2.37	1.87	2.33	2.29	2.49	0.19	8.33
Fluoxetine	0.10	5.19	5.64	6.15	4.27	5.41	5.19	5.89	0.71	13.63
Haloperidol	0.01	1.37	1.72	1.80	1.23	1.55	1.37	1.76	0.39	28.50
Lithium carbonate	0.32	0.63	0.38	1.32	0.66	0.50	0.63	0.85	0.22	35.35
Midazolam	0.00	0.00	0.00	0.00	0.00	0.00	0.00	0.00	0.00	0.00
Nortriptyline	0.03	0.52	0.61	0.70	0.46	0.56	0.52	0.65	0.13	25.10
Sodium valproate	0.50	0.60	0.73	0.85	0.67	0.66	0.60	0.79	0.19	31.72

DDD: Defined Daily Dose; SUS: Unified Health System.

**Figure 3:**
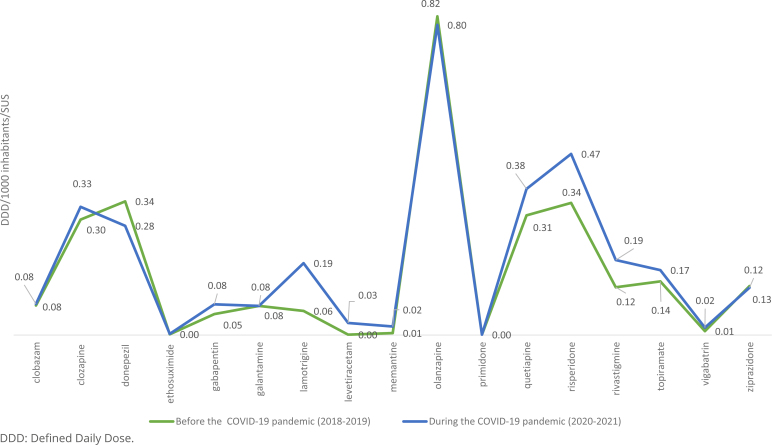
Consumption in Defined Daily Dose/1000 inhabitants/day of psychotropic drugs in the Specialized Pharmaceutical Services Component in the Unified Health System of Minas Gerais, 2018-2021.

The analysis of dispensation records of CEAF showed that olanzapine was the most dispensed drug in 2020-2021 (mean DDD=0.80), followed by risperidone (mean DDD=0.47) and quetiapine hemifumarate (mean DDD=0.38) (Figure 3). The analysis of the historical series pointed to a significant statistical difference between the two evaluated periods (p=0.019), with increasing consumption of psychotropic drugs dispensed in CEAF. The increasing consumption presented high percentage rates for levetiracetam (3000%) and memantine hydrochloride (340%), observing the reduced consumption for the active ingredients donepezil (-18.31%), ziprasidone (-3.71%) and olanzapine (-2.68%) (Table 2).

**Table 2:** Comparison of the consumption of psychotropic drugs in the Specialized Pharmaceutical Services Component in Minas Gerais, 2018–2021.

Active ingredient	DDD/1000 habitantes/dia/SUS									
	2018	2019	2020	2021	Mean	Median	Before the COVID-19 pandemic (2018-2019)	During the COVID-19 pandemic (2020–2021)	Absolute variation	Percentage variation (%)
Clobazam	0.05	0.11	0.03	0.13	0.08	0.08	0.08	0.08	0.00	5.26
Clozapine	0.28	0.32	0.34	0.32	0.31	0.32	0.30	0.33	0.03	11.11
Donepezil	0.34	0.35	0.31	0.26	0.31	0.32	0.34	0.28	-0.06	-18.31
Ethosuximide	0.00	0.00	0.00	0.00	0.00	0.00	0.00	0.00	0.00	50.00
Gabapentin	0.05	0.06	0.08	0.08	0.07	0.07	0.05	0.08	0.03	46.30
Galantamine	0.07	0.08	0.08	0.07	0.08	0.08	0.08	0.08	0.00	0.00
Lamotrigine	0.04	0.09	0.20	0.17	0.12	0.13	0.06	0.19	0.12	198.39
Levetiracetam	0.00	0.00	0.01	0.05	0.02	0.01	0.00	0.03	0.03	3000.00
Memantine	0.00	0.01	0.02	0.03	0.01	0.01	0.01	0.02	0.02	340.00
Olanzapine	0.75	0.89	0.76	0.84	0.81	0.80	0.82	0.80	-0.02	-2.68
Primidone	0.00	0.00	0.00	0.00	0.00	0.00	0.00	0.00	0.00	0.00
Quetiapine	0.29	0.33	0.38	0.37	0.34	0.35	0.31	0.38	0.07	22.08
Risperidone	0.27	0.41	0.44	0.50	0.40	0.42	0.34	0.47	0.13	37.06
Rivastigmine	0.10	0.15	0.19	0.19	0.16	0.17	0.12	0.19	0.07	56.91
Topiramate	0.14	0.13	0.17	0.17	0.15	0.16	0.14	0.17	0.03	21.01
Vigabatrin	0.01	0.01	0.02	0.02	0.01	0.02	0.01	0.02	0.01	90.00
Ziprasidone	0.12	0.13	0.13	0.11	0.12	0.13	0.13	0.12	0.00	-3.17

DDD: Defined Daily Dose; SUS: Unified Health System.

## DISCUSSION

This study showed increasing consumption of psychotropic drugs dispensed both in the scopes of CBAF and CEAF in SUS-MG, between 2018 and 2021. The analysis per periods demonstrates there was significant growth in the consumption of psychotropic medicines during the pandemic (2020-2021) for most of the analyzed active ingredients. Fluoxetine hydrochloride and diazepam were the most consumed psychotropic drugs in SUS, Minas Gerais, during the COVID-19 pandemic, which is a similar profile to other regions, in which the most consumed medicine are antidepressants and anxiolytics^13,20,21^. When we observe consumption, before and during the COVID-19 pandemic, the highest percentage values were verified for the active ingredients clonazepam and haloperidol (CBAF) and levetiracetam and memantine hydrochloride (CEAF). In the international context, the findings of this study are compatible with some locations that registered an increasing consumption of psychotropic drugs in the first year of the pandemic^22,23^, although some dissonant studies did not point out to changes in the profile of psychiatric prescriptions in the services^24^.

In the beginning of the COVID-19 pandemic, in 2020, an increasing prevalence of depressive and anxiety disorders was observed, associated to the higher rates of infection by the SARS-Cov-2 and several measures to face the pandemic, which reduced social interactions^4,25^. Besides the fear of becoming ill, for many people the COVID-19 pandemic caused a feeling of insecurity regarding health, the social and the economic spheres^26^. Feelings of anxiety, sadness or trouble sleeping are understandable answers to the social changes resulting from the pandemic, and should be understood as a way of social suffering, and not as symptoms of mental disorders^27^. Therefore, it was essential for the health professionals involved in this process to be able to distinguish common symptoms that were inherent to this period from the symptoms of a pathology on course. Despite the acute growth of mental health symptoms, there was a reduction in the subsequent months, thus becoming indistinguishable from the pre-pandemic symptoms in most population subgroups in 2020^28^. Therefore, the discussion about changes in the consumption profile of psychotropic drugs should also include other factors and changes that took place in health services in this period^11^, which can be related to the changes in this profile, as well as future actions for the continuity of care addressed to users.

In this study, even though the growing consumption of anxiolytics and antidepressants can be associated with the context of uncertainties and concerns generated by the pandemic^25^, there are other questions to be addressed referring the increasing consumption of chronic mental health medications. During the COVID-19 pandemic, two important changes that affected health policies can help to discuss the increasing number of dispensations during this period, especially for chronic mental disorders. First, the change in legislation that regulates the dispensation of psychotropic drugs through RDC resolution n. 357, from March 24, 2020, extended until September, 2023^12^, which temporarily increased the maximum quantities of medication allowed in controlled prescriptions and notifications and allowed remote delivery, defined by a specific public program, and delivery in the household of these medicines. It is observed that this resolution tripled the number of dispensed drugs in a single prescription, and allowed remote delivery, which was then forbidden in the country, to facilitate the access at a time of social and economic fragility for a great part of the population.

Secondly, we observed the increasing transfer of financial resources to cities, through Ordinance n. 2,516, from the Ministry of Health, for the acquisition of medicines in CBAF; the justification were the social impacts caused by the COVID-19 pandemic^10^. This financial transfer occurred only in 2020, but given the deadlines for the acquisition of medicines, its execution was also observed in 2021, allowing the amplified offer of medicines to the population through SUS. It is important to mention that, with these changes, the Psychosocial Support Network (RAPS) was already facing significant changes in the past years, initiated by the publication of ordinances and resolutions that altered the structure of the services network. Such changes influenced the access and the services provided to users, and these actions were considered by some authors as a dismantling of RAPS, due to the withdrawn of resources and the backward step in the network disposition^29^. Besides, several health services adopted closure measures or changes in the routine of services, and even the suspension or reorganization of activities during the pandemic. This situation may have impacted the access to proper care even more.

The observation of these factors not only reveals a multicausal view to analyze the increasing dispensation of psychotropic drugs during the pandemic, but also a very worrisome context in the scope of mental health policy. At a time of extreme vulnerability of the population, the access and offer of these medications is facilitated while the service was compromised, be if due to the fragility of RAPS or the general overload of the services and professionals in the first years of the pandemic. Because of the high potential for dependence and abuse of psychotropic drugs observed in the past few years, these changes, even if transient, can be in charge of altering behaviors that will certainly require continuous work from Pharmaceutical Services focused on the rational use of medicines and, in more complex cases, multidisciplinary work in demedicalization practices^17^.

The increasing dispensation of drugs can also be related to the access to new technologies in SUS. In the scope of the specialized component, there was increasing dispensation of levetiracetam, which is possibly not related to an epidemiological matter, but instead, of access to new technologies for treatment. In 2017, levetiracetam was incorporated in SUS to treat refractory epilepsy and patients with microcephaly^30^, so, the expressive growth in its dispensation in the subsequent years also reflects the diffusion of the drug in SUS. This is important data extracted from the databases to understand the time it takes for a technology to be implemented in clinical practice; the process still presents many barriers and challenges, once the time reported in some studies for the technology to be available in the services is much longer than the 180 days recommended in the current legislation^31^.

In this context, it is necessary to highlight the initiatives to encourage the use of databases in the records of dispensation of Pharmaceutical Services. The National Database of Pharmaceutical Services and Actions, in the Unified Health System (BNAFAR) gathers epidemiological and pharmaceutical services data all over the country, enabling an integrated view of the information^13^. The incentives to use BNAFAR were intensified with the growing number of legal instruments to regulate the use and sharing of data among the three federated entities, establishing that administrators became obligated to send the data after 2016^32^, as well as to provide the homologated portal to administrators in 2018^33^.

In Minas Gerais, SIGAF is a centralized and official system to manage the consumption of controlled and antimicrobial products in public services, so it is an important base for BNAFAR. The increasing records of dispensation after 2018 can be associated with the implementation of the BNAFAR portal^33^ and to actions that encourage the use of that base^34^. In the last public notices of the National Program of Pharmaceutical Services Qualification in the Unified Health System (QUALIFAR-SUS)^35^, the transfers prioritized the cities that were already registered in the program, that is, the ones that fulfill some requirements, such as the regularity in sending data of dispensation to BNAFAR, for example.

This study analyzed a secondary database, used mostly in the administrative scope, subjected to several sources of errors and limitations. Besides the sources of errors inherent to the entry and registration of data, such as typos, the analyzed reports did not allow the crossing of some records, thus preventing the checking or conference of some information. Several sources of bias were identified, related to the classifications inserted by the administrators and unmeasured confounding factors, or even the different environments and multiple users of the system. Even though the legislation establishes the obligatoriness of accounting for psychotropic drugs and SIGAF is the official database for this entry in Minas Gerais, some cities do not often update the system or choose to use another base to control primary care medicines; therefore, this database is subjected to underreporting of dispensation records, especially for the list of medicines in CBAF. Still, we highlight that the extracted data include an opportunity to use administrative data to indicate tendencies in the consumption of medicines, once there are no systematically organized clinical records that can compile this data in the scope of primary and outpatient care.

The change in the profile of dispensation of psychotropic drugs during the COVID-19 pandemic provides data for the management of services. Therefore, further studies are important to complement, confirm or rule out this identified profile and establish new comparisons and tendencies taken on in the post-pandemic context. The profile analysis of psychotropic drugs use becomes essential to monitor and improve access and care policies addressed to users, contributing with better mental health conditions for the Brazilian population and the rational use of medicines.
